# Improving Water Resistance and Mechanical Properties of Crosslinked Waterborne Polyurethane Using Glycidyl Carbamate

**DOI:** 10.3390/polym16192794

**Published:** 2024-10-01

**Authors:** Yong Rok Kwon, Jun Ho Park, Hae Chan Kim, Seok Kyu Moon, Dong Hyun Kim

**Affiliations:** 1 User Convenience Technology R&D Department, Korea Institute of Industrial Technology (KITECH), 143, Hanggaul-ro, Sangnok-gu, Ansan-si 15588, Gyeonggi-do, Republic of Korea; yongrok@kitech.re.kr (Y.R.K.); park4944@kitech.re.kr (J.H.P.); coolskawk@kitech.re.kr (H.C.K.); anstjrrb@kitech.re.kr (S.K.M.); 2 Department of Material Chemical Engineering, Hanyang University, 55, Hanggaul-ro, Sangnok-gu, Ansan-si 15588, Gyeonggi-do, Republic of Korea; 3 Department of Applied Chemistry, Hanyang University, 55, Hanggaul-ro, Sangnok-gu, Ansan-si 15588, Gyeonggi-do, Republic of Korea

**Keywords:** waterborne polyurethane, crosslinking, water resistance, glycidyl carbamate, coating

## Abstract

Waterborne polyurethane (WPU) often suffers from poor water resistance and mechanical properties due to hydrophilic emulsifiers. To address these issues, this study introduces glycidyl carbamate (GC) as a crosslinker to improve WPU performance. Three types of GC were synthesized using aliphatic, cycloaliphatic, and aromatic isocyanates, respectively. The crosslinked network was established through a reaction between the epoxide group of GC and the carboxylic acid and amine groups of WPU. Among these, the WPU film utilizing aromatic isocyanate-based GC exhibited the highest crosslink density, modulus, hardness, and water resistance, due to the rigidity of the aromatic molecular structure. However, the film displayed excessive brittleness, resulting in reduced tensile strength, along with yellowing typically associated with aromatic compounds. The WPU crosslinked with cycloaliphatic GC demonstrated the next best mechanical properties and water resistance, with a 2.7-fold increase in tensile strength, a 1.5-fold increase in hardness, and a 66% reduction in the water swelling ratio compared to neat WPU. This study presents a novel and effective strategy to enhance the water resistance and mechanical properties of WPU films, making them suitable for advanced coating applications.

## 1. Introduction

Amid the ever-increasing regulation of volatile organic compound (VOC) emissions, waterborne polyurethanes (WPUs) have received considerable attention over the past few decades [1,2,3]. WPU is a binary colloid in which polyurethane particles are dispersed in a continuous aqueous medium. These green systems do not produce VOCs, because only water evaporates during the process. WPUs achieve dispersion stability in the aqueous phase through the integration of an internal emulsifier into the main chains [4]. However, the presence of the internal emulsifiers adversely affects the water resistance of WPU films and coatings [5].

Several methods have been reported to improve the water resistance of WPUs, such as hydrophobic modifications and the introduction of nanofillers. Naghash et al. improved the water resistance and antifouling properties of WPU coatings by modifying them using hydroxyl-terminated polydimethylsiloxane with low surface energy [6]. Zhang et al. confirmed that the introduction of a fluorinated chain extender effectively reduced the surface energy of WPU coatings by concentrating fluorine atoms on the coating surface [7]. However, these hydrophobic segments may reduce the dispersion stability and mechanical strength of the WPU. The nanofillers not only prevent water permeation by improving the compactness and barrier properties of the WPU coating, but also greatly enhance the mechanical and thermal properties. In a recent study, WPU nanocomposites with improved water resistance were prepared by loading SiO_2_, TiO_2_, ZnO, carbon nanotubes, graphene oxide, and montmorillonite [8,9,10,11,12,13]. Nevertheless, the decrease in the transmittance of WPU coatings due to the introduction of nanofillers remains a problem.

Chemical crosslinking is an effective method for improving the water resistance of WPU films. Lei et al. crosslinked WPU particles using polyfunctional amines during chain extension. As a result, the water resistance of the WPU film increased with the degree of crosslinking [14]. Zhang et al. prepared UV-curable waterborne polyurethane acrylate (WPUA) polymers by end-capping a PU prepolymer with acrylate, and investigated the effect of the average functionality of WPUA on its properties. They found that an increase in the average functionality improved the water resistance and tensile strength of WPUA-cured films [15]. Although the crosslinking of WPU clearly improves its water resistance and mechanical properties, the presence of permanent hydrophilic groups can adversely affect the properties of the corresponding films and coatings.

Wang et al. demonstrated that the glycidyl group can serve as an effective functional group to introduce crosslinking structures in WPU [16]. In this work, we prepared a glycidyl carbamate (GC) that can chemically combine with the carboxyl group of dimethylol propionic acid (DMPA), which is used as an internal emulsifier in WPU. GC with a urethane group had excellent compatibility with WPU, and a water-resistant crosslinked WPU coating was designed using it as a crosslinker. To the best of our knowledge, no previous studies on crosslinking WPU with a GC-based crosslinker have been reported. The effect of GC content on the WPU coating was systematically investigated. These attempts effectively improved the water resistance and mechanical properties without compromising the optical properties of the WPU coating.

## 2. Experiment

### 2.1. Materials

Poly(tetrahydrofuran) (PTHF, *M*_n_ = 1000 g/mol, Sigma Aldrich, St. Louis, MI, USA) and DMPA (Sigma Aldrich, USA) were dried in a vacuum oven at 60 °C for 24 h before use. Isophorone diisocyanate (IPDI), tolylene-2,4-diisocyanate (TDI), hexamethylene diisocyanate (HDI), glycidol, dibutyltin dilaurate (DBTDL), trimethylamine (TEA), and ethylene diamine (EDA), supplied by Sigma Aldrich (USA), were used as received.

### 2.2. Synthesis of Glycidyl Carbamate (GC) with Different Diisocyanates

**Figure 1:**
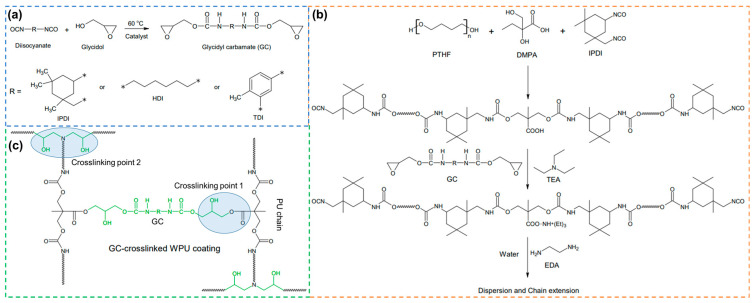
Synthetic schematic diagram of (**a**) GC with different diisocyanates and (**b**) WPUs containing GC, and (**c**) the chemical structure of GC-crosslinked WPU coatings.

A synthetic schematic diagram of GCs prepared with various diisocyanates is shown in Figure 1a. Diisocyanate (HDI, IPDI, or TDI, 0.03 mol), glycidol (0.07 mol), and THF were added to a 100 mL three-necked round-bottom flask. The mixture was stirred at 60 °C until a clear solution was achieved. As an exception, the GC samples in which TDI was used were synthesized in a cold water bath due to its high reactivity. DBTDL was then introduced as a catalyst, and the reaction was allowed to proceed until the disappearance of the NCO peak, as confirmed by Fourier transform infrared (FTIR) spectroscopy. The resulting product was thoroughly washed with deionized water (10 times) to remove residual glycidol, and then dried in a vacuum oven at 60 °C for 24 h. Table 1 shows the yield, epoxy equivalent weight, and characteristics of GCs synthesized by the different diisocyanate types.

### 2.3. Preparation of WPU Dispersions with GCs

The synthesis pathway of WPU is shown in Figure 1b. PTHF (0.05 mol), DMPA (0.025 mol), and IPDI (0.1 mol) were added to a 500 mL four-necked flask equipped with a mechanical stirrer, reflux condenser, and thermometer. The reaction was performed for 2 h in a nitrogen atmosphere at 80 °C in the presence of DBTDL. The dibutylamine back-titration method confirmed that the NCO% of the polyurethane prepolymer reached the theoretical value, and the temperature inside the reactor was reduced to 40 °C. Then, while maintaining this temperature, TEA (0.025 mol) was added to neutralize the polyurethane prepolymer for 1 h. After neutralization and cooling the reactor to room temperature, GCs with different diisocyanate types were added so that the molar ratio between the carboxyl group of DMPA and the epoxy group of GC was 1:1. Then, setting the mechanical stirrer to 600 rpm, deionized water and EDA (0.025 mol) were slowly added dropwise over 20 min, and stirring was continued for 1 h. The products were a blue clear dispersion with a solid content of 30%. The final WPUs were named WPU-I, WPU-H, and WPU-T, based on the initials of the diisocyanate used in the synthesis of GC.

### 2.4. Preparation of GC-Crosslinked WPU Films

First, WPU-I, WPU-H, and WPU-T were each cast on a Teflon plate and dried at 60 °C for 6 h to remove water. The dried films were then cured at 120 °C for 1.5 h. The resulting WPU films were separated from the Teflon plate and stored in a desiccator prior to analysis. The chemical structure of the GC-crosslinked WPU coatings are shown in Figure 1c.

### 2.5. Characterization

#### 2.5.1. Gel Fraction

The gel fraction of the WPU films was evaluated by solvent extraction. The films were repeatedly reflux-extracted in tetrahydrofuran for 4 h by a Soxhlet extractor. The undissolved films were collected, rinsed with tetrahydrofuran, and then dried in a vacuum oven for 24 h at 40 °C. Then, the dried insoluble films were weighed [17]. The gel fraction was calculated using Equation (1):

Gel fraction = ω_1_/ω_0_ × 100 (1)

where ω_1_ and ω_0_ are the weights of the dried insoluble film and initial film, respectively.

#### 2.5.2. FTIR Spectroscopy

The attenuated total reflection–Fourier transform infrared (ATR–FTIR) spectra of WPU dispersions and films were recorded with a Cary 630 spectrometer (Agilent Tech., Inc., Santa Clara, CA, USA) in the wavenumber range of 4000–650 cm^−1^ with a resolution of 4 cm^−1^.

#### 2.5.3. Transmission Electron Microscopy (TEM)

The morphology of dispersion particles was characterized on a Tecnai F20 G2 (FEI, Hillsboro, OR, USA) instrument. Before measurement, the dispersion was stained with a 3 wt% phosphotungstic acid solution and then dried on 200 mesh copper.

#### 2.5.4. Particle Size Distribution

The size of the WPU dispersions and the corresponding distributions were measured using a laser diffraction particle size analyzer (LS 13 320, Beckman Coulter, Brea, CA, USA). All samples were diluted in distilled water to 0.1% [18].

#### 2.5.5. Thermal Analysis

Thermogravimetric analysis (TGA) was performed on a TA-2000 instrument (Dupont Co., Wilmington, NC, USA) under a N_2_ flow of 20 mL min^−1^, at temperatures ranging from 30 to 600 °C and a heating rate of 10 °C min^−1^.

#### 2.5.6. Dynamic Mechanical Analysis (DMA)

The dynamic mechanical behaviors of the prepared films were characterized using a DMA7100 (Hitachi High-Tech, Tokyo, Japan) with tensile mode with a frequency of 1 Hz in the temperature range of −80 to 120 °C at a heating rate of 5 °C min^−1^.

#### 2.5.7. Mechanical Properties Measurements

The tensile properties of the WPU films were measured using a universal testing machine (UTM, 5ST, Tinius Olsen, Horsham, PA, USA) with a crosshead speed of 10 mm min^−1^. A rectangular specimen of 40 × 10 mm^2^ (length × width) was used for analysis.

Nano-indentation tests (ZHN, Zwick Roell, Ulm, Germany) were conducted to assess the mechanical properties of the surface of the WPU films, which were prepared with a thickness of 40 μm on glass substrates. For each sample, ten indentations were performed, and the corresponding load–displacement curves were recorded to determine the hardness and elastic modulus.

#### 2.5.8. Swelling Ratio

The swelling ratio of the WPU films was measured by immersing the dried WPU coating into distilled water at 20 °C. After that, the swelled films were dried in a vacuum oven until the mass remained constant. The swelling ratio of the films was calculated using Equation (2):

Swelling ratio = ((ω_1_ − ω_0_))/ω_0_ × 100, (2)

where ω_1_ and ω_0_ are the weights of the swollen and dried film, respectively.

#### 2.5.9. Water Contact Angle (WCA) Measurements

The WCAs of the WPU film surfaces were measured with the sessile drop method at room temperature, using a telescopic goniometer (OCAH-200, DataPhysics, Filderstadt, Germany) equipped with a video capture device. A 5–10 μL volume of distilled water was pumped onto the surfaces of the films using a microsyringe, and images were captured using the telescopic device fitted with a video camera. Each WCA value was expressed as the average value of five independent measurements, taken at different locations of the same film.

## 3. Results and Discussion

### 3.1. Characterization of Structures for the GCs and WPU

**Figure 2:**
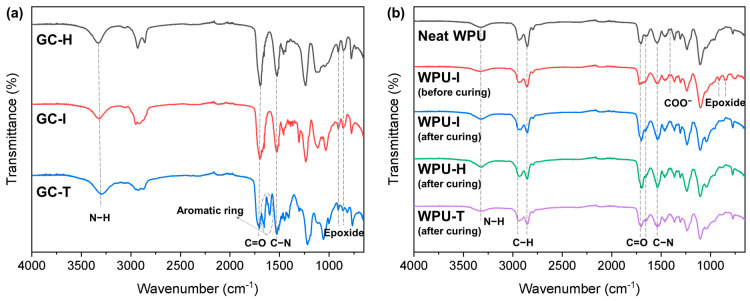
FTIR spectra of (**a**) GCs with different diisocyanates and (**b**) neat WPU, uncured WPU-I, cured WPU-I, cured WPU-H, and cured WPU-T films.

Figure 2a presents the FTIR spectra of GC-H, GC-I, and GC-T. Notably, no peaks corresponding to the NCO group were observed in the 2270–2240 cm^−1^ range across all GC samples [19]. The spectra displayed characteristic peaks associated with the carbamate group at 3320 cm^−1^ (N–H stretching), 1698 cm^−1^ (C=O stretching), and 1524 cm^−1^ (C–N stretching) [19]. Signals indicative of the epoxide group were identified at 908 cm^−1^ (asymmetric stretching) and 858 cm^−1^ (symmetric stretching) [20]. For GC-T, peaks corresponding to aromatic C=C stretching vibrations were detected within the 1660–1600 cm^−1^ range [21].

Figure 2b shows the FTIR spectra of neat WPU, uncured WPU-I, and cured WPU-I. The WPU samples exhibited characteristic peaks associated with urethane and urea groups at 3322 cm^−1^ (N–H stretching), 1708 cm^−1^ (C=O stretching, urethane), 1648 cm^−1^ (C=O stretching, urea), and 1541 cm^−1^ (C–N stretching) [19]. Additionally, signals corresponding to the symmetric and asymmetric C–H stretching vibrations of the CH_2_ group were observed at 2930 and 2854 cm^−1^, respectively. The C–O–C symmetric and asymmetric bands were also identified within the wavenumber range of 1047–1161 cm^−1^ [22].

The FTIR spectrum of the uncured WPU-I sample revealed two distinct peaks at 912 and 854 cm^−1^, corresponding to the epoxide rings [20]. In the cured WPU-I sample, the peaks corresponding to the epoxide groups and the COO^−^ peak at 1412 cm^−1^ disappeared [23]. This indicates that the epoxide group of GC-I reacted with the amine and carboxylic acid groups in WPU. Similar consumption of the carboxyl and epoxide groups was noted across all cured WPU samples, confirming that GC, with its approximately two epoxide groups per molecule, effectively forms a crosslinked structure within the WPU matrix.

### 3.2. Particle Size and Morphology of WPU Dispersions

**Figure 3:**
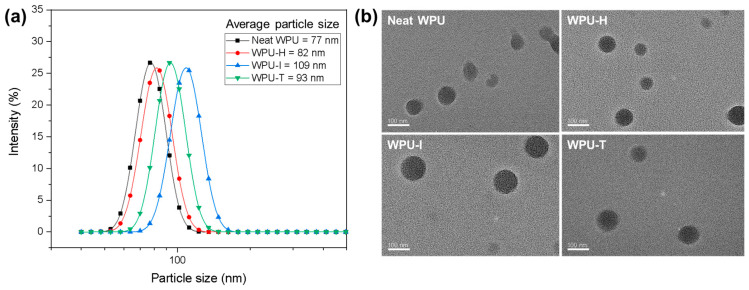
(**a**) Particle size distributions and (**b**) TEM images of WPU dispersions.

Figure 3a shows the particle size distribution of the prepared WPU dispersions. The particle sizes of the GC-integrated WPU dispersions were larger than those of the neat WPU. This increase in particle size can be attributed to the relative hydrophobicity of GC compared to the PU chains containing carboxylate ions, which makes GC’s self-stabilization in water difficult. Consequently, GC is likely emulsified within the WPU particles with the assistance of the hydrophilic segments of PU. The particle size distributions across all WPU samples exhibited a narrow and single peak, suggesting that GC was uniformly distributed within the WPU matrix. The particle size order for WPU containing GC followed the sequence WPU-I > WPU-T > WPU-H. This trend is related to the amount of GC contained in the WPU. Due to the different epoxy values of the GC samples, 5.8, 5.1, and 5.0 wt% of GC-I, GC-T, and GC-H were added to the WPU, respectively. As the content of hydrophobic GC increased, the particle size of the WPU also tended to increase. Despite this, the average particle size of all WPU dispersions remained below 120 nm, ensuring a stable dispersed phase even after 6 months of storage.

Figure 3b presents a TEM image of the WPU dispersions, revealing that the dispersions exhibit a well-defined spherical morphology. The observed size variations in the TEM image are consistent with the particle size measurement results, further confirming the trends identified in the particle size analysis.

### 3.3. DMA Properties of WPU Films with Different GCs

**Figure 4:**
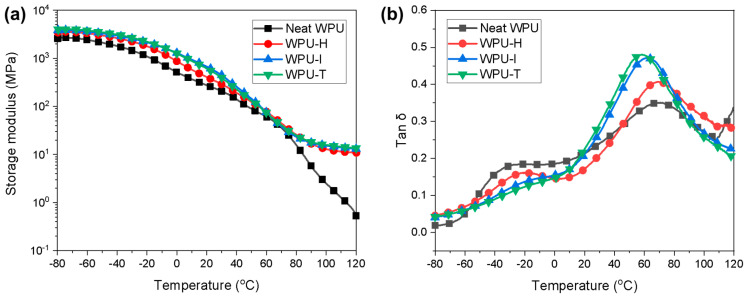
(**a**) Storage modulus and (**b**) tan *δ* of WPU films with different GCs as a function of temperature.

The variation in the storage modulus of WPU films as a function of temperature is illustrated in Figure 4a. The GC-crosslinked WPU samples exhibited higher modulus values across all temperature ranges compared to the neat WPU, with a rubbery plateau observed above 100 °C. This behavior can be attributed to the classic stiffening effect induced by crosslinking [24]. Typically, the molecular rigidity follows the order of aromatic (GC-T) > alicyclic (GC-I) > aliphatic (GC-H), and this trend is clearly reflected in the DMA results [25]. However, within the 20 to 50 °C range, WPU-I demonstrated the highest modulus, likely due to the good compatibility and strong hydrogen bonding between the IPDI-based WPU and the GC-I crosslinker.

Figure 4b presents the loss coefficient (tan *δ*) curves of the WPU samples, which provide insights into the glass transition temperature (*T_g_*) and phase separation behavior [26]. The *T*_g_ values of the WPU samples, determined from the tan *δ* peaks, are summarized in Table 2. All samples exhibited two distinct *T*_g_ values: the lower temperature *T*_g_ corresponds to the soft segment (*T*_gss_), while the higher temperature *T*_g_ corresponds to the hard segment (*T*_ghs_). The difference between *T*_ghs_ and *T*_gss_ (*ΔT*_g_) was reduced in the crosslinked WPU samples compared to the neat WPU, which can be attributed to the phase mixing effect induced by crosslinking.

The crosslink density of WPU-H, WPU-I, and WPU-T was calculated from the storage modulus in the rubbery region using the following equation:

*ν*_e_ = *E*′/3*RT* (3)

where *ν*_e_ represents the crosslink density in mol/cm^3^, *E*′ is the elastic storage modulus in the rubbery region of the crosslinked WPU film, R is the ideal gas constant, and *T* is the absolute temperature at which *E*′ was measured [24]. The crosslink densities (*ν*_e_) of WPU-T and WPU-I were found to be nearly identical, while the *ν*_e_ of WPU-H was relatively lower (Table 2). This lower crosslink density may be attributed to the increased flexibility of WPU-H, which results from the longer alkyl chains of GC-H.

### 3.4. Gel Fraction and Appearance of WPU Films

The crosslinking reaction between the epoxide group of GC and the carboxyl group of WPU was facilitated by the tertiary amine (TEA) used as a neutralizing agent. Traditionally, crosslinked WPUs are synthesized using a pre-cure method involving trimethylolpropane to form crosslinked particles [27]. However, this approach often limits the achievable crosslink density due to gelation during WPU synthesis, and it may weaken the bonding forces at the particle interfaces when forming the final film. In contrast, the crosslinked WPU developed in this study employs a post-cure method, where crosslinking occurs after film formation, thereby overcoming the limitations associated with the pre-cure method.

**Figure 5:**
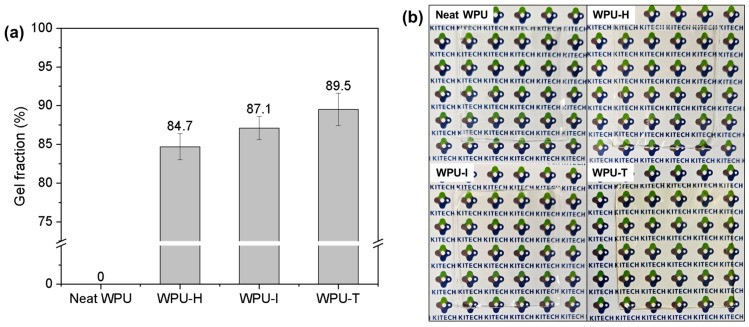
(**a**) Gel fraction and (**b**) film appearance of WPU samples with different GCs.

Figure 5a presents the gel fractions of neat WPU, WPU-H, WPU-I, and WPU-T. The neat WPU was fully soluble in the organic solvent, whereas the gel fraction of the GC-crosslinked WPU samples followed the order WPU-T > WPU-I > WPU-H. This order is consistent with the crosslink density trend calculated from the DMA results.

WPU-T exhibited the highest modulus and crosslink density, attributed to the inherent rigidity of GC-T. However, the presence of benzene groups in GC-T imparted a yellowish hue to the final coating (Figure 5b). This yellowing effect can pose a limitation in applications requiring clear coatings, potentially restricting the use of WPU-T in such contexts.

### 3.5. TGA Measurement

**Figure 6:**
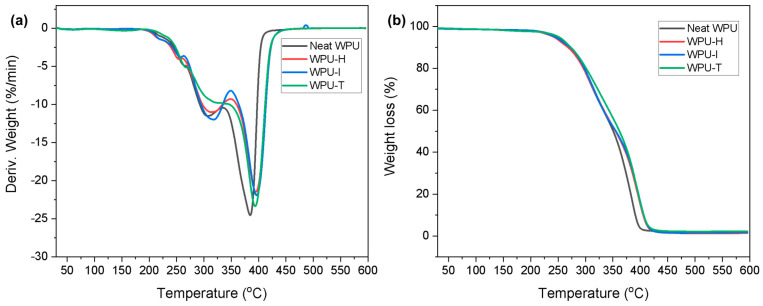
(**a**) DTG and (**b**) TG curves of WPU films crosslinked with different GCs.

The derivative thermogravimetry (DTG) and TG curves of cured WPU films containing different GCs are presented in Figure 6. The DTG curve reveals two distinct peaks corresponding to maximum weight loss. The first peak is attributed to the pyrolysis of the urethane and urea groups, which constitute the hard segment of the WPU. The second peak represents the decomposition of the soft segments [28]. The GC-crosslinked WPU samples exhibited maximum weight loss at higher temperatures compared to neat WPU (Figure 6a). Specifically, the first peak temperatures for neat WPU, WPU-H, WPU-I, and WPU-T were 307 °C, 313 °C, 316 °C, and 324 °C, respectively. This trend is consistent with the crosslink density results obtained from DMA and gel fraction measurements, indicating that a higher degree of crosslinking requires more energy to restrict molecular chain movement and disrupt the structure [29]. In other words, the increased crosslink density in the WPU films resulted in improved thermal stability. Notably, WPU-T, which incorporates a benzene group known for its high thermal stability, demonstrated the smallest weight loss across all temperature ranges (Figure 6b).

### 3.6. Water Resistance

**Figure 7:**
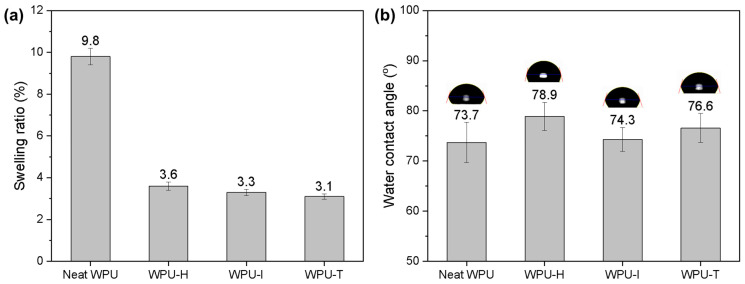
(**a**) Swelling ratio and (**b**) water contact angle of WPU films crosslinked with different GCs.

The swelling ratio and water contact angle are key indicators of water resistance and are crucial properties for evaluating the performance of coatings. Figure 7 presents the swelling ratio and water contact angle of WPU films crosslinked with different GCs. The swelling ratio of GC-crosslinked WPU was reduced by up to 68.4% compared to neat WPU, owing to the formation of a denser polymer network that effectively hinders the penetration of water molecules. This reduction is also due to the consumption of carboxylic acid and amine groups in WPU during the crosslinking reaction. The swelling ratios followed the trend WPU-T < WPU-I < WPU-H, which is consistent with the crosslink density and the rigidity of the crosslinker. A polymer matrix with greater flexibility allows more water to permeate because the network can more easily rearrange itself in response to environmental changes.

As shown in Figure 7b, neat WPU exhibited the lowest water contact angle, which can be attributed to the presence of strong hydrophilic functional groups in the uncrosslinked WPU. Interestingly, the water contact angle measurements for the GC-crosslinked WPU samples did not follow the same trend as the swelling ratios. WPU-H showed the highest water contact angle, likely due to the high flexibility of GC-H, which may have facilitated the migration of hydrophobic segments to the film–air interface during the film formation process [30].

### 3.7. Mechanical Properties

**Figure 8:**
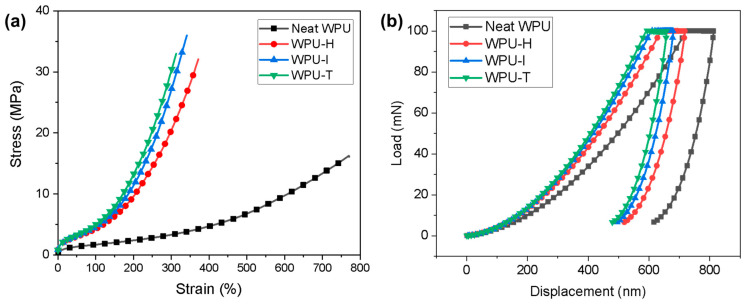
(**a**) Tensile stress–strain curves and (**b**) load–indentation depth curves of WPU films with different GCs.

Figure 8a shows the stress–strain curves of non-crosslinked and crosslinked WPU films. As outlined in Table 3, the modulus (at 100% elongation) and tensile strength of WPU films crosslinked with GCs showed an increase, whereas the elongation at break exhibited a decrease. This phenomenon can be attributed to the crosslinking effect, which restricts the mobility of polymer chains during tensile deformation, thereby enhancing mechanical strength. The tensile strengths at the breaking point for WPU-H, WPU-I, and WPU-T were measured to be 32.1, 36.0, and 33.0 MPa, respectively. The modulus (at 100% elongation) followed the order WPU-T > WPU-I > WPU-H, which is attributable to the molecular rigidity of the GCs utilized. However, the tensile strength at the breaking point for WPU-I was higher than that of WPU-T, likely due to the greater brittleness observed in WPU-T.

Figure 8b illustrates the load–nano-indentation depth curves, which reflect the mechanical surface behavior of WPU samples applied to glass substrates. The hardness (HIT), elastic modulus (EIT), and brittleness index are presented in Table 3. HIT, representing the material’s resistance to plastic deformation, is influenced by the stiffness of its bonding network. In contrast, EIT measures the material’s resistance to elastic deformation and is related to the strength of atomic bonds [31]. HIT and EIT were calculated using the following equation:

*HIT* = *F*/*A_p_* and *EIT* = (*1 − υ*^2^)/[*1*/*E_r_ −* (*1 − υ_i_*^2^)/*E_i_*] (4)

Here, *F* represents the maximum applied force, and *A_p_* refers to the projected contact area. The Poisson ratios of the sample and the indenter are denoted by *ν* and *ν_i_*, respectively. Additionally, *E_r_* signifies the reduced modulus of the indentation contact, and *E_i_* corresponds to the elastic modulus of the indenter [31].

The incorporation of GC imparted a crosslinked structure to the WPU matrix and increased the proportion of urethane groups. Consequently, the HIT and EIT values for WPU samples crosslinked with GCs were elevated, indicating improved resistance to surface penetration. These trends align well with the modulus @100% observed in the tensile properties. Notably, the brittleness index (HIT/EIT) was highest for WPU-T, corresponding to its low elongation in the tensile tests.

## 4. Conclusions

The effects of GC-H, GC-I, and GC-T, synthesized with HDI, IPDI, and TDI, respectively, on the mechanical properties and water resistance of crosslinked WPU were systematically evaluated. The addition of GC, which exhibits greater hydrophobicity compared to WPU, resulted in a slight increase in the particle size of WPU. The incorporation of GC introduced covalent chemical bonds and abundant urethane groups into the WPU network, leading to significant enhancements in mechanical properties such as modulus and hardness. Notably, WPU-T, which achieved the highest modulus (5.0 MPa at 100% elongation) and hardness (101.8 MPa), showed the best overall rigidity but also displayed yellowing, limiting its application in transparent coatings. Among the samples, WPU-I recorded the highest tensile strength of 36.0 MPa. Furthermore, the water swelling ratio was reduced by up to 68%, reflecting enhanced water resistance due to the crosslinking with GC. These quantitative improvements suggest that GC-crosslinked WPU films are highly promising for applications requiring enhanced mechanical strength and water resistance. Despite these promising results, the high cost of isocyanate limits its potential for large-scale applications. To overcome this, future research could explore hybrid formulations with cost-effective polymers to reduce material costs while maintaining performance, thereby expanding the application scope of WPU coatings in cost-sensitive industries.

## Figures and Tables

**Table 1:** Properties of GCs with different diisocyanate types.

Name	Diisocyanate Type	Yield (%)	Epoxy Value (mol/100 g)	Appearance
**GC-H**	HDI	91.2	0.61	Viscous, colorless
**GC-I**	IPDI	95.8	0.53	High viscosity, colorless
**GC-T**	TDI	96.4	0.60	High viscosity, yellow

**Table 2:** *T*_g_ and *ν*_e_ of WPU films with different GCs.

Name	*T*_gss_ (°C)	*T*_ghs_ (°C)	*ΔT*_g_ (°C)	*ν*_e_ (mol/cm^3^)
**Neat WPU**	−25.8	70.7	96.5	-
**WPU-H**	−20.1	69.8	89.9	1.11 × 10^−3^
**WPU-I**	−14.4	62.5	76.9	1.37 × 10^−3^
**WPU-T**	−14.1	58.1	72.2	1.38 × 10^−3^

**Table 3:** Mechanical properties of WPU films with different GCs.

Name	Modulus (@100%, MPa)	Tensile Strength (MPa)	Elongation at Break (%)	HIT (MPa)	EIT (GPa)	Brittleness Index (HIT/EIT)
**Neat WPU**	1.7 ± 0.1	16.1 ± 1.4	770 ± 56	60.8 ± 2.3	2.8 ± 0.2	0.022 ± 0.001
**WPU-H**	4.3 ± 0.3	32.1 ± 2.3	371 ± 24	85.0 ± 2.7	3.7 ± 0.2	0.023 ± 0.001
**WPU-I**	4.6 ± 0.3	36.0 ± 2.1	341 ± 19	94.1 ± 2.8	4.0 ± 0.2	0.024 ± 0.001
**WPU-T**	5.0 ± 0.3	33.0 ± 2.5	313 ± 22	101.8 ± 3.1	4.1 ± 0.3	0.025 ± 0.001

## Data Availability

The data are available upon request.
